# The amplification of α-synuclein amyloid fibrils

**DOI:** 10.1042/BST20250489

**Published:** 2026-04-30

**Authors:** Alexander K. Buell

**Affiliations:** Department of Biotechnology and Biomedicine, Technical University of Denmark, 2800 Kgs. Lyngby, Denmark

**Keywords:** alpha-synuclein, amplification, amyloid, fragmentation, secondary nucleation, seeding

## Abstract

Amyloid fibrils formed by α-synuclein are a hallmark of a range of neurodegenerative diseases, notably Parkinson’s disease, multiple system atrophy (MSA), and dementia with Lewy bodies, collectively known as synucleinopathies. Recent years have seen an increasing understanding of the structural architecture and diversity of α-synuclein amyloid fibrils. Furthermore, our mechanistic understanding of the formation of these structures has also experienced significant progress. Here, I provide a concise overview of the current state of knowledge of how α-synuclein amyloid fibrils can be amplified, i.e., increase in number. The main emphasis is thereby on the process of secondary nucleation, i.e., the generation of new amyloid fibrils catalyzed by existing fibrils. A detailed understanding of fibril amplification is relevant in the context of the spread of pathology in the central nervous system of synucleinopathy patients. In addition, it can also be exploited in the framework of diagnostic approaches collectively known as seed amplification assays (SAAs). In such assays, the minute quantities of α-synuclein fibrils present in biological fluids are amplified and possibly quantified for disease diagnostics.

## Introduction

Amyloid fibrils are non-covalent homo-molecular protein polymers with very high aspect ratios, i.e., very long and thin structures. Amyloid fibrils are extensively studied due to their involvement in biological functions and diseases [1]. Individual amyloid fibrils consist of several tens up to several thousand individual protein monomers that are arranged hierarchically. They form so-called protofilaments, chains with cross-sections composed of individual molecules, which then often assemble further into higher-order helical structures, i.e., mature amyloid fibrils [2]. The formation of amyloid fibrils from purely soluble protein can be thought of as a nucleation event akin to the formation of a crystal seed and is also found to be associated with a significant free energy barrier [3]. However, when the initial barrier has been overcome and an amyloid fibril is present, it can grow through the templated addition of individual protein monomers to the ends of the fibrils, a process that is found to be orders of magnitude faster than nucleation [4,5]. Furthermore, it has been found in many cases that amyloid fibrils can be amplified effectively under certain conditions. The generation of new fibrils from existing ones can occur through fragmentation of the existing amyloid fibrils, which generates new growth-competent ends [6–8]. This process is generally assumed not to depend on the concentration of soluble protein. Alternatively, the formation of new fibrils can explicitly depend on the presence of soluble protein in what is known as secondary nucleation [9]. Secondary nucleation corresponds to the nucleation of a new fibril from soluble protein but catalyzed by the presence of an existing fibril.

Recent years have seen significant progress in our understanding of the amplification of amyloid fibrils formed by the protein α-synuclein, and here I set out to summarize this progress. The active interest in amyloid fibril amplification stems from its role in both disease propagation and disease diagnostics. The finding that the aggregate pathology tends to spread throughout the central nervous system (CNS) as the disease progresses in most neurodegenerative disorders, including the synucleinopathies [10], suggests that amyloid fibrils are being amplified inside patients’ CNS. Therefore, inhibiting amyloid fibril amplification could be an effective way to hinder or slow down disease progression. Furthermore, amyloid fibril amplification is also being exploited actively in so-called seed amplification assays (SAAs) [11]. The presence of α-synuclein aggregates in biological fluids (notably CSF, but also blood and saliva) is a characteristic of synucleinopathies, but the concentrations of such amyloid fibrils are too low to be easily and directly detectable. If they can be amplified through the addition of recombinant soluble protein substrate, they can be detected more readily and thereby be used as a diagnostic marker.

## The amplification of amyloid fibrils

The formation, growth, and amplification of filamentous protein aggregates, such as those formed by actin, tubulin, or insulin, have been studied for several decades, and it is well established that mechanical action, such as shear forces or sonication, can fragment protein filaments [12]. To this day, many biophysical experiments of amyloid fibril formation are performed in the presence of mechanical shear forces, which induces fragmentation and therefore amplification of the fibrils. This effect is also exploited in the amplification of amyloid fibrils for diagnostic purposes in Protein Misfolding Cyclic Amplification [13], whereby prion amyloid fibrils are repeatedly fragmented by ultrasound and allowed to grow by addition of recombinant substrate.

In the 1980s, it was found by Ferrone et al. that filamentous aggregates of sickle hemoglobin can amplify through secondary nucleation on the surface of existing filaments, a process that they demonstrated to depend very strongly on the concentration of soluble protein [14].

Secondary nucleation and fragmentation of protein filaments, collectively referred to as secondary processes, are therefore usually conceptualized as two distinct phenomena. The rate of secondary nucleation is proposed to depend on both the concentration of fibrillar and soluble protein, whereas fragmentation rates do not depend on the soluble protein concentration. On the other hand, fragmentation depends very strongly on the presence of mechanical forces that actively break the fibrils, whereas secondary nucleation is observed under relatively quiescent solution conditions.

In 2008, it was proposed that insulin amyloid fibrils are able to amplify through such a surface-catalyzed secondary nucleation process [15]. In a series of landmark theoretical papers, Cohen et al. developed an analytical theoretical framework to define the mechanisms of filament amplification from kinetic data sets of filament formation [16]. This theoretical advance allowed global analytical fits of large kinetic data sets acquired at various protein concentrations and led shortly after its development to the discovery that amyloid fibrils formed by the amyloid-β peptide also amplify through secondary nucleation [17], i.e., through a process the rate of which depends on both the concentration of soluble protein as well as the mass/surface of available fibrils. Apparently, the surface of amyloid fibrils comprises in some cases particularly favorable sites for the nucleation of additional filaments. It has recently been proposed that these sites correspond to defects in the underlying fibril core structure [18].

These important discoveries of amyloid fibril amplification through secondary nucleation led to a systematic search as to the generality of such a monomer-dependent amplification process and, notably, whether such a process also contributed to the amplification of α-synuclein amyloid fibrils.

## The secondary nucleation of α-synuc lein amyloid fibrils

The experimental strategy to probe for the existence of secondary nucleation in α-synuclein aggregation needed to be different from the one that had been employed for the amyloid-β peptide. While the latter forms fibrils very effectively from purified soluble protein, α-synuclein aggregates very slowly, especially in the absence of active mechanical perturbation [8,19]. Furthermore, the primary nucleation of α-synuclein amyloid fibrils is thought to occur at hydrophobic–hydrophilic interfaces, such as the air–water interface [19] or polymer–water interfaces [20]. Control over the aggregation conditions therefore also requires taking the material and surface properties of the reaction vessel into account. Often, protein-repellant multiwell plate surfaces are chosen in order to suppress surface-induced primary nucleation, even though this effect can even be inverted in the presence of certain compounds [21]. The slow aggregation of α-synuclein under such conditions often leads to poorly reproducible data sets, preventing the straightforward analysis of quiescent unseeded aggregation data with respect to the presence or absence of secondary nucleation. The solution to these difficulties consists of weakly seeded experiments, i.e., experiments in which the number of seed fibrils is sufficient to lead to measurable aggregation, but insufficient to convert the entire amount of soluble protein into amyloid fibrils without fibril amplification events. This can be illustrated by assuming a seed concentration of 10 nM (in monomer equivalents) in the presence of 100 μM soluble monomer. In order to consume all the available monomer, each seed fibril needs to grow therefore on average to 10 000 times its initial length. Sonicated seed fibrils of ca. 100 nm length would therefore theoretically need to grow to 1 mm in length. Such weakly seeded experiments thereby lead to amyloid fibrils of a very high surface-to-end ratio and facilitate the detection of secondary processes. A systematic screen of different seed concentrations and pH values led to the discovery that α-synuclein amyloid fibrils were indeed able to amplify in the absence of active shaking with a rate that depended very strongly on the pH [8,22]. While it was difficult to detect any amplification at neutral pH in these initial experiments at moderate protein concentrations and ionic strengths, mildly acidic pH showed very pronounced amplification, with an estimated increase in amplification rate of about 4 orders of magnitude from pH 7 to pH 5.5 [8]. This strong pH-dependence of fibril amplification is in stark contrast with that of fibril elongation that was found to vary by less than an order of magnitude over the same pH range. The strong pH dependence of this amplification that was immediately attributed to secondary nucleation plausibly stems from the C-terminal tail of α-synuclein that is disordered in both the monomeric and the fibrillar state. The high density of carboxylic acid functional groups leads to a significant upshift of the pKa values compared with the isolated amino acids and explains why pH changes in the mildly acidic range can have an effect on the protonation state of the C-terminal residues [23]. This conclusion is supported by the finding that C-terminally truncated variants of α-synuclein that lack a significant fraction of the negative charges of the WT protein display secondary nucleation at more neutral pH values [24].

**Figure 1:**
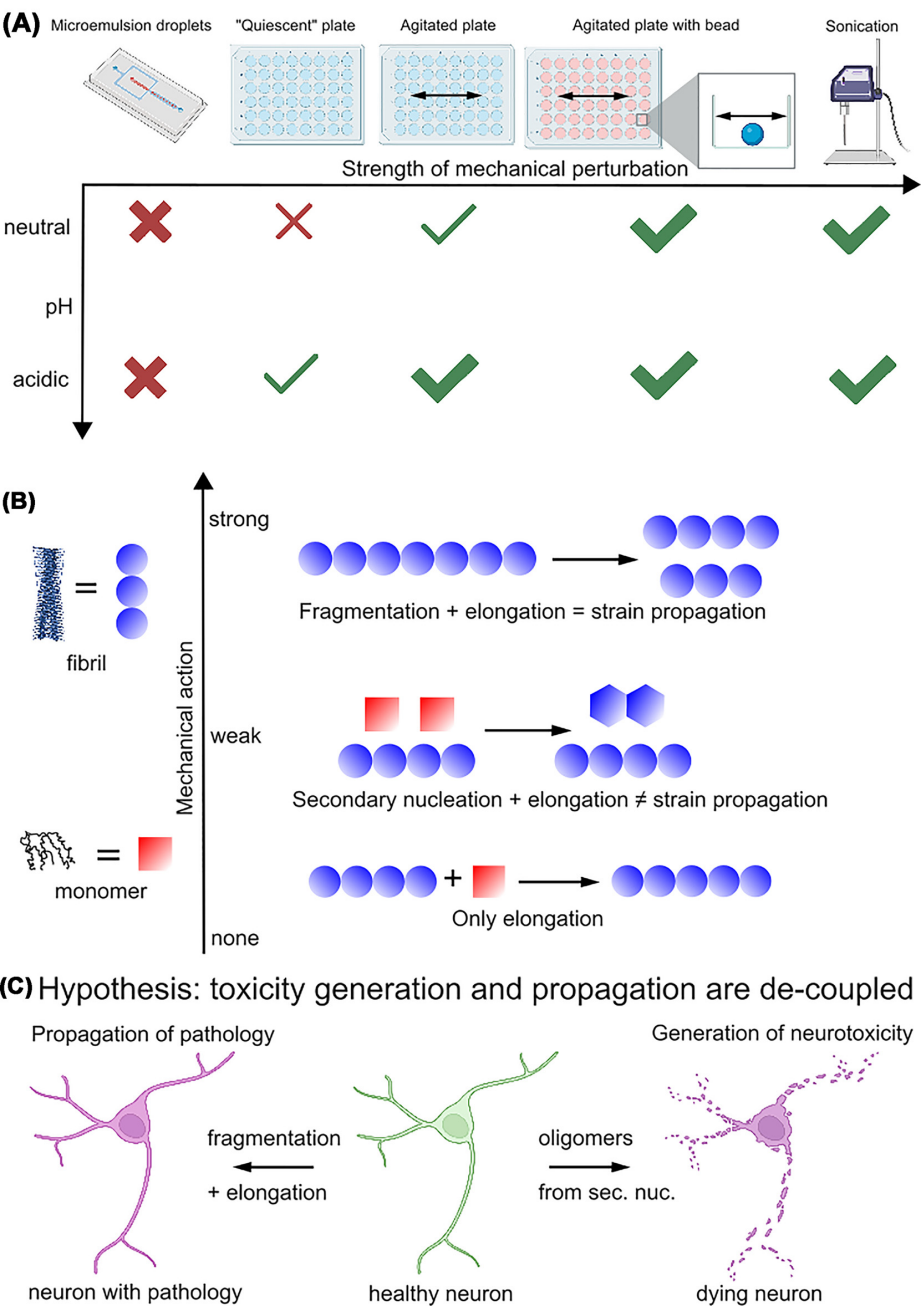
The mechanisms and biological consequences of α-synuclein fibril amplification (**A**) Amyloid fibrils of α-synuclein can be amplified through mechanical action, whereby the sensitivity to mechanical perturbation increases at acidic pH compared with neutral pH. No fibril amplification is observed under entirely quiescent conditions in micron-scale compartments. (**B**) Fibril amplification under weak mechanical perturbation occurs mostly through secondary nucleation, which does not appear to be able to propagate the structural information of the seed fibrils. Strong mechanical action leads to fibril fragmentation, which, in conjunction with the growth of the fibril fragments through monomer addition, does in most cases propagate the structure of the seed fibril. (**C**) Given the evidence that fibril structure is propagated inside affected organisms but that at the same time, toxic aggregate species appear to be generated by secondary nucleation, I propose a working hypothesis: both fragmentation and secondary nucleation can occur in affected organisms, whereby the former leads to the propagation of pathology and the latter to the generation of neurotoxicity.

It has been demonstrated through a range of experimental methods, notably surface-based biosensing [22] and nuclear magnetic resonance [23,25,26], that monomeric α-synuclein can interact with fibril surfaces, which are decorated by a “fuzzy coat” of the N-terminal and C-terminal sequence regions that are not part of the β-sheet fibril core. This interaction features a strong pH dependence that mirrors that of the pH dependence of secondary nucleation itself [22,27], providing additional evidence for this pH-dependent amplification process being indeed secondary nucleation on fibril surfaces. At neutral pH, the affinity of this interaction, which is mediated by the N-terminus of the soluble monomer and the C-terminally disordered tail of the protein inside the fibrils, is very weak (mM range) [23]. The weak affinity of soluble α-synuclein for fibril surfaces at neutral pH probably explains the inefficiency of secondary nucleation under these conditions (Figure 1a). It has, however, been demonstrated that secondary nucleation can be observed even at neutral pH [28], in particular at the very high concentrations present in biomolecular condensates of α-synuclein [29], which are in the mM range. Some direct microscopic evidence for association of soluble α-synuclein with fibril surfaces and the subsequent formation of new fibrils by secondary nucleation has also been acquired by two-color super-resolution microscopy [7].

An important question in the context of secondary nucleation is whether the structural properties of the seed fibrils are preserved in a similar manner to what is generally accepted for fibril elongation [30]. Amyloid fibrils are highly polymorphic and this is particularly true for α-synuclein. Its polypeptide chain can fold into a multitude of different structures upon assembly and often several different fibril structures are observed under a given set of solution conditions [31]. It has mostly, even though not exclusively, been found that the growth of amyloid fibrils through monomer addition results in the incoming monomer adopting the same structure as the template fibril (Figure 1b). However, it was found that this is not necessarily the case for secondary nucleation. Neutral and mildly acidic pH conditions lead to different fibril structures, and it was shown that weakly seeded experiments, whereby a solution of monomers at a given pH was seeded with fibrils made at a different pH, led mostly to fibrils of a structure dictated by the conditions under which they are forming rather than a structure imposed by the seed fibrils [31,32]. In other words, the structural information of the seed fibrils is not preserved in such cases (Figure 1b). This lack of structural propagation by secondary nucleation may be able to explain the lack of faithful propagation of the fold of fibrils extracted from the brains of multiple system atrophy (MSA) patients reported in one study [33]. The latter experiments were performed under conditions that are likely to favor secondary nucleation and fibril growth appears to be inefficient in these propagation experiments. Most new fibrils may have formed through secondary nucleation, which is also consistent with the resulting fibrils resembling those formed *de novo* under the same conditions [31]. Surface-based secondary nucleation therefore does not appear to be able to override the natural tendency of α-synuclein to fold into a given fibril structure dictated mostly by the solution conditions. Strongly seeded experiments with short fibrils, on the other hand, are found to lead to a propagation of the fibril structure, confirming that templated growth occurs [32].

Interestingly, it has been observed that sequence variants of α-synuclein, e.g., disease-related single-point mutations [34,35], as well as charge variants [36], can modulate the secondary nucleation rates dramatically. The latter study also provides evidence that different seed fibril morphologies feature differences in the secondary nucleation rate under otherwise identical conditions [36]. Similarly, post-translational modifications that change the charge state of α-synuclein, such as N-terminal acetylation [35] and phosphorylation [37], can be expected, and are indeed found in some cases [35], to modify the secondary nucleation rate. Such results, while individually difficult to interpret, may represent a small window onto the detailed molecular mechanism of secondary nucleation of α-synuclein. Additional systematic mutagenesis studies will be necessary in order to fully rationalize these findings and put them into a larger mechanistic context.

## Other sources of α-synuclein fibril amplification

While it is becoming increasingly obvious that secondary nucleation plays an important role in the amplification of α-synuclein and other types of amyloids under some conditions, other processes can also contribute to the amplification of α-synuclein fibrils. Mechanical effects, such as agitation of the sample in the presence or absence of beads, as well as sonication, can effectively enhance fibril fragmentation and therefore accelerate the aggregation rate [6,8,38]. Ultrasonication has been shown to break fibrils into short fragments of the order of 100 nm in length or below [6,7], increasing seeding potential very significantly. Mechanical shaking, on the other hand, whether in the presence of beads or not, is usually less efficient in breaking the fibrils. This difference in fragmentation efficiency is demonstrated by the observation that when sonicated fibrils are exposed to shaking in the presence of beads, the kinetics are only accelerated after the fibrils have grown for a considerable amount of time and have therefore reached a length at which they are indeed susceptible to the effects of the moving beads [8].

The fragmentation of mature α-synuclein fibrils in the complete absence of any mechanical effects has not yet been directly demonstrated, presumably because thermal fluctuations at moderate temperatures are not sufficient to lead to spontaneous breakage of such fibrils at detectable rates. However, the chaperone system Hsp70–DNAJB1–Apg2 has been demonstrated to be able to fragment α-synuclein amyloid fibrils under ATP-dependent energy consumption [39]. While this tri-chaperone system mainly leads to monomer removal from the ends of fibrils, fragmentation has also been observed, thereby enhancing the seeding potential of the fibrils [39,40]. Furthermore, it has recently been shown that the treatment of surface-bound α-synuclein amyloid fibrils with the non-specific proteinase-K leads to an increase in seeding efficiency in biosensing experiments [27]. The protease treatment initially leads to the removal of the “fuzzy coat” of the fibrils, presumably followed by damage to the β-sheet core of the fibrils. Eventually, this treatment may lead to partial or full fragmentation of the fibrils and thereby an increase in the number of growth-competent ends.

## Exploiting α-synuclein fibril amplification for diagnostic applications

The highly successful diagnostics of prion diseases with SAAs [13] have prompted the development of such assays also for other amyloid diseases, notably for the synucleinopathies [11,41]. SAAs are based on the addition of recombinant substrate protein to biological fluids containing minute quantities of seed fibrils. Repeated cycles of growth and amplification of the fibrils lead eventually to a strong enough amyloid dye fluorescence signal to be detected in a standard fluorescence microwell plate reader. Fibril amplification is usually achieved through cycles of sonication [13] or strong mechanical agitation in the presence of beads [41] that induces the fragmentation of the fibrils. One major limitation of such assays is that they are not very quantitative, in the sense that samples with very different seed concentrations display very similar kinetic curves, due to the strong amplification of the seeds that “erases” most of the memory of the underlying initial seed concentrations. Digital SAAs have been proposed as a solution to render these assays more quantitative. There, the sample is divided into thousands of micron-scale compartments, such as water-in-oil emulsion droplets [42], micron-sized wells, or microgels [43]. Ideally, the sample is diluted to such an extent that each micro-compartment either contains a seed or no seed. If sufficient amplification can be achieved, then the compartments that initially contained a seed will end up containing thousands of fibrils and thereby become fluorescent, and the ones that were devoid of any seeds will remain dark. This digital (light versus dark) behavior ultimately allows the initial seed concentration to be counted and thereby quantified. The strong confinement of such compartments makes it challenging to apply the common methods of fibril amplification through mechanical agitation. Therefore, the use of acidic pH appears to be a good option for the amplification of the seeds through secondary nucleation inside such compartments. Under such conditions, efficient amplification of α-synuclein amyloid fibrils is observed in multiwell plates without active shaking. However, no fibril amplification, but only elongation of the seed fibrils, was detected inside microcompartments [42,43]. This difference in behavior is surprising because the solutions in the wells of the non-binding plates had been additionally covered with the same fluorinated oil/surfactant combination that is used to generate the microdroplets, such as to eliminate as much as possible the influence of plate surface and air–water interface and make the two setups highly comparable [42]. It was subsequently found that the movement of the plate during the reading cycles represented a sufficient mechanical perturbation to lead to efficient amplification at acidic pH in plates. Therefore, kinetic experiments in microwell plates that are generally considered to be quiescent, i.e., without active shaking, are in fact not completely free of mechanical perturbations. It can be concluded from these experiments that some level of mechanical perturbations is required in order to enable secondary nucleation to lead to fibril amplification [28,42]. The complete quiescence inside microdroplets is therefore not conducive to fibril amplification (Figure 1a). This conclusion is corroborated by the observation that subjecting water-in-oil microdroplets to mechanical agitation through a piezo shaker led to detectable fibril amplification [42].

Taken together these observations suggest that both fibril fragmentation and secondary nucleation require mechanical perturbation, with the latter requiring much weaker levels of such perturbations compared with the former (Figure 1a). Secondary nucleation therefore depends on the protein concentration and ionic strength, and very sensitively on solution pH and the degree of mechanical agitation. At mildly acidic pH, it is observed at moderate protein concentrations and without active shaking [8,22], whereas neutral pH requires high protein concentrations (such as, e.g., encountered in biomolecular condensates [29]) or very high ionic strength [28], or else active shaking [44]. It therefore appears that both the binding of the monomer to the fibril surface (favored by low pH and high ionic strength and protein concentration) as well as presumably the removal of the secondary nuclei from the fibril surfaces (favored by mechanical agitation) are required for efficient secondary nucleation.

## Inhibition of secondary nucleation

The important role that secondary nucleation of α-synuclein amyloid fibrils may play in the generation of toxic oligomers (Figure 1c) has led to efforts to develop effective inhibitors of this process. Several different types of inhibitors were identified. One of the most effective inhibitors of the secondary nucleation of amyloid-β, the Brichos chaperone [45], has also been demonstrated to be able to inhibit the secondary nucleation of α-synuclein. It has been observed that Brichos can undergo one-dimensional diffusion along the fibrils and thereby possibly brush off the surface-bound monomeric α-synuclein and inhibit secondary nucleation [46]. This proposed mechanism of Brichos is distinct from the one proposed for amyloid-β, whereby Brichos is believed to selectively bind to the defect sites where secondary nucleation occurs [18].

A protein that binds with high affinity to monomeric α-synuclein, AS69, has been reported to have highly sub-stoichiometric inhibitory action on α-synuclein secondary nucleation that cannot be explained through monomer sequestration alone [47]. Through the generation of a covalent construct between AS69 and α-synuclein, it was shown that it is not AS69 itself but the AS69-α-synuclein complex that is the inhibitory species in this case [48]. It was proposed that this complex interacts with secondary nuclei and prevents their further evolution into seeding competent species. Less efficient suppression of secondary nucleation that required stoichiometric inhibitor concentrations has been demonstrated with a d-enantiomeric peptide (SVD1), which had been identified from a mirror-image phage display screen against monomeric α-synuclein [49].

Other studies have shown that off-pathway oligomers [50] and small molecules [51] can also inhibit secondary nucleation of α-synuclein. Overall, the picture emerges that interaction with the fibril surface in general, or with the nucleation sites or the secondary nuclei specifically, can effectively suppress secondary nucleation. Which of the above-mentioned approaches will ultimately prove the most effective one to suppress toxicity generation and/or pathology spreading through secondary nucleation is not yet clear, given the different modes of action and CNS availabilities of these different inhibitor classes. Furthermore, additional examples for each inhibitor class may be needed in order to be able to decide which of them is most flexible and adaptable. However, interaction of the inhibitor with the fibril surface appears to be a more viable strategy than sequestration of the monomer, given the functional biological roles of α-synuclein.

## The role of secondary nucleation in synucleinopathies

Amyloid fibrils are often found to propagate their structural properties faithfully both *in vitro* and *in vivo* [52]. In particular, the finding that each synucleinopathy is characterized by a distinct set of fibril morphologies [53,54] suggests that the amplification of amyloid fibrils inside affected individuals preserves their structural properties. Faithful propagation of structural properties upon injection into mouse brains was recently demonstrated for fibrils generated *in vitro* that have structural similarity to the fibrils extracted from MSA patients [52]. At the same time, evidence from *in vitro* experiments suggests that surface-catalyzed secondary nucleation does not preserve the structure of the underlying seed fibril, if the conditions under which the secondary nucleation occurs favor a different fibril structure from the one of the seed fibrils. Therefore, it is not clear whether the spreading of α-synuclein pathology throughout the CNS of affected individuals [10] is caused by fibril amplification through secondary nucleation. If the cellular conditions are homogeneous throughout the pathway of spreading, the daughter fibrils might adopt the same structure despite not directly inheriting the structure of the seed fibrils. On the other hand, it is also possible that fibril amplification *in vivo* occurs through passive or active fragmentation processes, e.g., enabled by chaperones, in conjunction with elongation, which would naturally preserve the fibril structure. However, it has been proposed that α-synuclein oligomers [22,44] as well as overall toxicity [55] are generated through secondary nucleation. I therefore propose a working hypothesis consistent with the available data, namely that the spreading of pathology and the generation of toxicity are mechanistically decoupled in the case of α-synuclein (Figure 1c). Secondary nucleation generates small species that are highly diffusive and toxic, but may not be very stable under cellular conditions, especially if generated under mildly acidic solution conditions where secondary nucleation is most effective [8,32], as can be encountered in endosomes and lysosomes. On the other hand, fibril fragmentation, whether active or passive, leads to stable fragments that preserve and propagate the structural characteristics of the fibrils from which they originate.

## Perspectives

- *Importance of the field*: The amplification of α-synuclein amyloid fibrils plays an important role in the spread of disease pathology but can also be exploited for diagnostic purposes in SAAs.
- *Summary of the current thinking*: Amyloid fibrils of α-synuclein can amplify in number through fragmentation and secondary nucleation, whereby both processes require mechanical action—no fibril amplification is observed under complete quiescence. Secondary nucleation displays a strong pH dependence, with acidic pH being particularly conducive, because it reduces the negative charge density on the C-terminal region. Despite much progress, the detailed mechanism of secondary nucleation remains unresolved.
- *Future directions*: Additional studies, including extensive and systematic mutagenesis, in conjunction with kinetic experiments under different solution conditions are likely to allow filling the mechanistic knowledge gaps in the future.

## Open Access

Open access for this article was enabled by the participation of Danmarks Tekniske Universitet in an all-inclusive *Read & Publish* agreement with Portland Press and the Biochemical Society under an agreement with the Royal Danish Library.

## Abbreviations

CNS: central nervous system
MSA: multiple system atrophy
SAAs: seed amplification assays
